# Rental Housing Deposits and Health Care Use

**DOI:** 10.1001/jamahealthforum.2024.2802

**Published:** 2024-09-06

**Authors:** Margae J. Knox, Elizabeth A. Hernandez, Jennifer Ahern, Daniel M. Brown, Hector P. Rodriguez, Mark D. Fleming, Amanda L. Brewster

**Affiliations:** 1 Kaiser Permanente Northern California Division of Research, Oakland; 2 School of Public Health, University of California, Berkeley; 3 Contra Costa Health, Martinez, California; 4 Contra Costa County Department of Public Health, Martinez, California

## Abstract

**Importance:**

Housing deposits and tenancy supports have become new Medicaid benefits in multiple states; however, evidence on impacts from these specific housing interventions is limited.

**Objective:**

To evaluate the association of rental housing deposits and health care use among Medicaid beneficiaries receiving social needs case management as part of a Whole-Person Care (Medicaid 1115 waiver) pilot program in California.

**Design, Setting, and Participants:**

This cohort study compared changes in health care use among a group of adults who received a housing deposit between October 2018 and December 2021 along with case management vs a matched comparison group who received case management only in Contra Costa County, California, a large county in the San Francisco Bay Area. All participants were enrolled in health and social needs case management based on elevated risk of acute care use. Data analysis took place from March 2023 to June 2024.

**Exposure:**

Rental housing deposit funds that covered 1-time moving transition costs. Funds averaged $1750 per recipient.

**Main Outcomes and Measures:**

Changes in hospitalizations, emergency department visits, primary care visits, specialty care visits, behavioral health visits, psychiatric emergency services, or detention intakes during the 6 months before vs 6 months after deposit receipt. Changes 12 months before and after deposit receipt were examined as a sensitivity analysis.

**Results:**

Of 1690 case management participants, 845 received a housing deposit (362 [42.8%] <40 years old; 422 [49.9%] male) and 845 received case management only (367 [43.4%] <40 years old; 426 [50.4%] male). In adjusted analyses, deposit recipients had no statistically significant differential changes in health care use for any measure compared to participants who received case management alone. Twelve-month sensitivity analyses yielded consistent results.

**Conclusions and Relevance:**

In this cohort study, compared to case management only, housing deposits with case management were not associated with short-term changes in health care use. There may be other unmeasured health benefits or downstream benefits from greater case management engagement. States considering housing deposits as an expanded Medicaid benefit may need to temper expectations about short-term health care use impacts.

## Key Points

**Question:**

Is rental housing deposit funding, a new Medicaid benefit in California and other states, associated with short-term changes in hospital, emergency department, outpatient, and other health care use?

**Findings:**

In this cohort study of 1690 participants, deposit funding with case management was associated with similar reductions, but not differential changes, in health care use over 6 months relative to a matched comparison group that received case management only.

**Meaning:**

Further process metrics with longer-term evaluation should be considered to help identify change mechanisms and nonutilization–related health benefits.

## Teaser

This cohort study evaluates the association of rental housing deposits and health care use among Medicaid beneficiaries receiving social needs case management as part of a Medicaid 1115 waiver pilot program in California.

## Introduction

As health care payers and delivery systems devote increasing attention to social determinants of health, housing insecurity is a top concern.^1,2^ People experiencing homelessness experience 2- to 9-fold greater hospital and emergency department use compared to similar housed peers.^3^ General housing insecurity is also associated with greater emergency department and urgent care use.^4^ Hence, some state Medicaid programs are implementing new benefits to support housing needs. At least 4 states in the latest Medicaid 1115 demonstration waivers (2022-2027) are funding housing deposits and other 1-time transition/moving costs (eg, first month’s rent, utility activation fees, relocation expenses).^5^ At least 9 states plan to help beneficiaries secure and maintain housing through housing navigation or tenancy supports.^5,6^

Interventions that provide housing directly, such as permanent supportive housing or hotel placements, have been shown to reduce health spending and hospital utilization.^2,7,8,9^ Some evidence also indicates rental assistance is associated with lower odds of poor health, less psychological distress, and fewer unmet health care needs.^10,11,12,13^ However, to our knowledge, prior studies have not examined health care–based housing deposit funding with case management. As additional states consider similar Medicaid benefits, evidence is needed to inform expectations of changes in health care use.

This cohort study makes a key contribution by investigating housing deposit funding with case management navigation and tenancy support, as proposed in some current Medicaid 1115 waivers. All participants in this study could receive navigation and tenancy support from their case manager as part of a program for beneficiaries with high acute care risk. We compared deposit funding recipients to those with case management only across several health care services: inpatient admissions, emergency department visits, primary care visits, specialty care visits, behavioral health visits, psychiatric emergency services, and detention intakes. We compared health care use changes 6 months before and after deposit receipt vs a propensity score–matched group. We hypothesized that housing deposits would be associated with improved residential stability and general health, thereby decreasing inpatient and acute care services while maintaining or slightly increasing routine care visits, given deferred health care maintenance.

## Methods

### Study Design

The CommunityConnect case management program in Contra Costa County, California, administered housing deposits to select participants facing housing instability as part of California’s Whole-Person Care Medicaid waiver program (2016-2021). The program focused on complex health and social needs among high-risk/high-utilizing enrollees.^14^ Contra Costa County is a large county in the San Francisco Bay Area with more than 1.1 million residents and 220 000 Medicaid beneficiaries.^15,16^ Further details about Contra Costa’s CommunityConnect case management program are described in other publications.^17,18,19,20^

All case management participants were Medicaid beneficiaries. Program enrollment began in 2017 and occurred automatically each month based on an algorithm for high risk of hospitalization or emergency department visits. Housing deposit recipients were actively working with a case manager, had secured a rental lease or rental agreement, and had a source of income to continue ongoing rental payments. Deposit funding was administered on a rolling basis beginning in October 2018. In total, the program distributed $1.9 million in housing deposits over 3 years. The maximum amount allowed was $5000. The median (IQR) deposit amount was $1750 ($920-$2900). Recipients were primarily individuals who had long-term experiences of homelessness. Funds were often used to secure a single room in a shared unit given the region’s high-cost, competitive housing market. Deposits could also help recipients move to lower-cost housing after job loss or other circumstances. The county administered the program with 1 full-time personnel managing deposit funding applications, auditing, and payment distribution processes.

All participants, including those who did not receive housing deposits, received ongoing case management services. Specialist case managers included nurses, social workers, mental health specialists, housing specialists, and substance use counselors who were assigned up to 100 higher-acuity participants. Community health workers were assigned up to 250 lower-acuity patients and provided telephonic-only care. Interaction frequency varied based on participant interests and needs. Program guidelines recommended once-per-month contact from specialist case managers and every-other-month contact from community health workers. Case managers could help both groups coordinate health care needs, connect to other social needs resources, and navigate housing issues like landlord communication, bill pay setup, cleaning routines, and potential isolation.

The Contra Costa Regional Medical Center and Health Centers Institutional Review Committee approved study procedures. Informed consent was waived due to administrative enrollment processes. Reporting follows the Strengthening the Reporting of Observational Studies in Epidemiology (STROBE) reporting guidelines.^21^

### Analytic Sample

**Figure 1.**
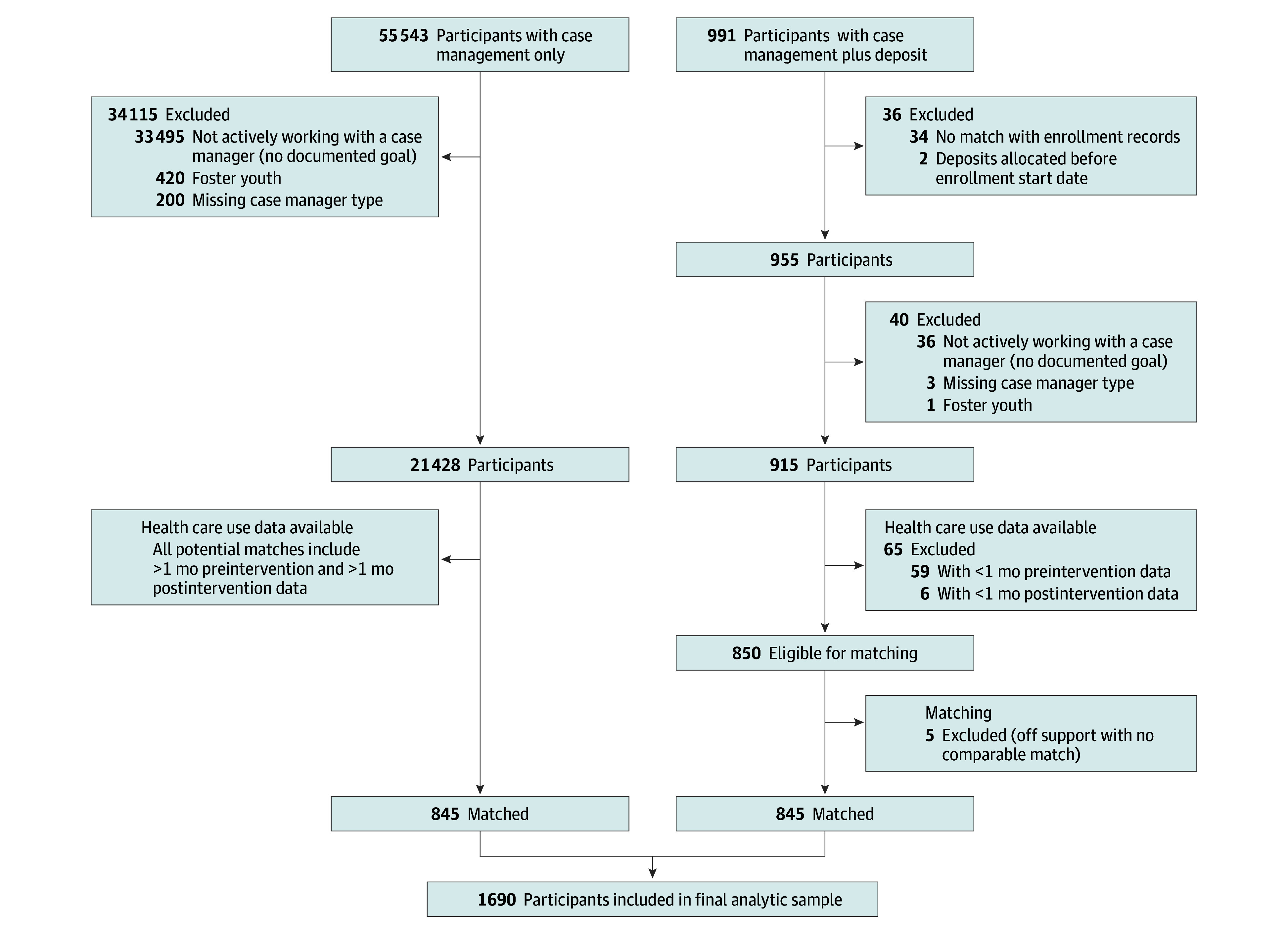
Participant Exclusions and Matching to Construct Final Analytic Sample

There were 991 housing deposit recipients from October 2018 to December 2021. Data analysis took place from March 2023 to June 2024. To be included in the analysis, deposit recipients must have been linkable to a case management record and have at least 1 goal documented, indicating work with a case manager, before deposit receipt. Deposit recipients with less than 1 month of preintervention data (n = 59) or less than 1 month of postintervention data (n = 6) were excluded. After matching, the total analytic sample included 1690 participants: 845 who received a move-in deposit and 845 with comparable characteristics who also worked with a case manager but did not receive a deposit (Figure 1).

### Matched Comparison Group

Housing deposit recipients were propensity score matched 1:1 using nearest neighbor matching with no replacement to identify similar case management participants who did not receive a move-in deposit. Although we assessed balance on observable characteristics, program requirements to secure a rental agreement and make ongoing rental payments may have produced unobservable differences like willingness to relocate neighborhoods or income stability. Limited local housing supply introduced some chance to deposit allocation, as housing may have become available in one month but not another. Case managers also accepted multiple sources of income documentation and, in relevant cases, worked with participants to apply for supplemental Social Security income. Matching characteristics are described further elsewhere in Methods.

Matches were paired within the same enrollment year to account for program maturity (eFigure 1 in Supplement 1). We calculated a counterfactual deposit date for each comparison group participant by adding to the comparison participant’s enrollment date the average enrollment to deposit lag for all deposit recipients enrolled in the same quarter.

### Data Structure

We summed counts of each outcome for the 6 months before and after the deposit date or calculated index date. Because outcome data were structured by month, the month that the deposit or index date occurred was not included in analysis to clearly delineate before and after time frames.

### Sensitivity Analyses

While longer time frames were of interest, extending the analysis from 6 to 12 months limited the sample. More than 12 months of outcome data were available for just 60% of participants preintervention and 83% postintervention, while more than 6 months of outcome data were available for 81% of participants preintervention and 94% postintervention. Thus, the primary specification examined outcomes 6 months before and after the deposit date or counterfactual date, and 12-month outcomes were examined as a sensitivity analysis. We also matched 5 comparison participants per deposit recipient with replacement as an additional sensitivity analysis.

### Outcomes

Outcomes included changes in counts of hospitalizations, emergency department visits, primary care visits, specialist visits, behavioral health visits, psychiatric emergency services, and jail (detention) intakes 6 months before and after deposit receipt vs before and after changes in the matched comparison group. Outcomes were identified from a data warehouse managed by the Contra Costa Health business intelligence team. The data warehouse documents relevant health care visits for all case management participants by combining Medicaid claims and electronic health records from the county-run hospital and a network of outpatient clinics.

Primary and specialty care visits included encounters with physicians (MD or DO) or nurse practitioners across the health system. Behavioral health visits include all visits with a marriage and family therapist, licensed clinical social worker, or psychologist. All outpatient encounters are distinct from visits with CommunityConnect case management personnel. Psychiatric emergency services included all admissions to a 23-bed facility for adult patients, the county’s only psychiatric emergency services unit. Jail intakes were documented because county health services provides health care for all county detention facilities.

### Covariates

Covariates used for matching represent participant characteristics that may affect both receiving a housing deposit and the trajectory of health care service use. Covariates were determined in part by comparing participants who received a housing deposit vs those working with a case manager who did not. Demographic covariates from patients’ electronic health records included sex, age (<40, 40-60, or >60 years), race and ethnicity (Asian or Pacific Islander, Black or African American, Hispanic or Latinx, White, or other or unknown [including American Indian or Alaska Native, other low-reported categories, and missing data]), and participants’ assigned case manager discipline (community health worker, nursing, social work, substance use counselor, or housing specialist).

Covariates from medical record documentation included histories of hypertension, diabetes, chronic obstructive pulmonary disorder, psychosis disorder, depressive disorder, alcohol or other drug dependence, behavioral health acuity (none, mild to moderate, or moderate to severe), detention history, and experience of homelessness.

Additional covariates included participant responses to housing screening questions that case managers asked all participants at the beginning of their case management enrollment. Housing questions included, “What is your current living situation?”; “Do you believe you are at risk of losing your housing in the next 6 months?”; “Would you like information about rental assistance resources?”; and “Would you like information about shelters in your area?” Last, a covariate was included to indicate whether program enrollment was automatic, based on predicted risk of future hospital or emergency department use, or a manual enrollment (eg, based on clinician referral).

### Statistical Analysis

Effect estimates were calculated using a difference-in-differences design, which examined the change in health care services use among individuals who received move-in deposits relative to the change in health care services use in the comparison group.^22^ Participants were matched on demographics, health measures, other relevant characteristics, and all preintervention health care use measures.

To examine the difference-in-differences assumption of preintervention parallel trends, we generated plots of health care use for each outcome (eFigure 2 in Supplement 1). The appearance of preintervention parallel trends was confirmed for each outcome by statistical tests for parallel trends at the *P* > .05 level.

We estimated the impact of housing deposits for each outcome by calculating both raw difference-in-differences and using negative binomial regression models with a group-by-time interaction. Model covariates included the same demographic, health, behavioral health, and housing screening question variables from the matching process. We then converted model estimates to marginal effects to derive average treatment effects for the study population. Negative binomial models were chosen due to the count distribution of the outcomes. All analysis was conducted using Stata, version 17 (StataCorp).

## Results

### Participant Characteristics

The final sample comprised 1690 case management participants, 845 of whom received a housing deposit and 845 of whom received case management only. Of participants who received a deposit in the final sample, 422 (49.9%) were male; 362 (42.8%) were younger than 40 years, 339 (40.1%) were aged 40 to 60 years, and 144 (17.0%) were older than 60 years; and 26 (3.1%) were Asian or Pacific Islander, 276 (32.7%) were Black or African American, 126 (14.9%) were Hispanic or Latinx, 336 (39.8%) were White, and 81 (9.6%) were other or unknown race or ethnicity. Additionally, 132 participants (15.6%) had moderate to severe behavioral health acuity, 491 (58.1%) had a history of alcohol and other drug dependence, 242 (28.6%) had homeless status documented in their medical record, and 392 (46.4%) believed that they were at risk of losing housing within 6 months. After matching, differences between the 2 groups were not statistically significant for all covariates, indicating that the groups were well matched on observable characteristics (Table 1).

**Table 1.** Characteristics Among the Housing Deposit Group and Comparison Group Before and After Matching

Characteristic	No. (%)					
	Before matching			After matching		
	Comparison group (n = 21 428)	Deposit group (n = 850)	*P* value	Comparison group (n = 845)	Deposit group (n = 845)	*P* value
Demographics						
Case manager type						
Community health worker	12 362 (57.7)	158 (18.6)	<.001	169 (20.0)	158 (18.7)	.99
Community health worker, specialist	1019 (4.8)	29 (3.4)		29 (3.4)	29 (3.4)	
Nursing professional	3193 (14.9)	128 (15.1)		134 (15.9)	127 (15.0)	
Social worker	1301 (6.1)	62 (7.3)		62 (7.3)	62 (7.3)	
Mental health professional	1270 (5.9)	50 (5.9)		48 (5.7)	50 (5.9)	
Housing specialist	658 (3.1)	104 (12.2)		102 (12.1)	102 (12.1)	
Substance use counselor	1625 (7.6)	319 (37.5)		301 (35.6)	317 (37.5)	
Sex						
Female	13 375 (62.4)	424 (49.9)	<.001	419 (49.6)	423 (50.1)	.85
Male	8053 (37.6)	426 (50.1)		426 (50.4)	422 (49.9)	
Age, y						
<40	8204 (38.3)	362 (42.6)	<.001	367 (43.4)	362 (42.8)	.92
40-60	8043 (37.5)	342 (40.2)		331 (39.2)	339 (40.1)	
>60	5181 (24.2)	146 (17.2)		147 (17.4)	144 (17.0)	
Race and ethnicity						
Asian or Pacific Islander	2166 (10.1)	26 (3.1)	<.001	27 (3.2)	26 (3.1)	.95
Black or African American	4766 (22.2)	280 (32.9)		285 (33.7)	276 (32.7)	
Hispanic or Latinx	6919 (32.3)	126 (14.8)		121 (14.3)	126 (14.9)	
White	5693 (26.6)	336 (39.5)		339 (40.1)	336 (39.8)	
Other or unknown^a^	1877 (8.8)	82 (9.6)		73 (8.6)	81 (9.6)	
Language used						
English	15 932 (74.4)	810 (95.3)	<.001	787 (93.1)	805 (95.3)	.06
Other language	5496 (25.6)	40 (4.7)		58 (6.9)	40 (4.7)	
Medical and personal history						
Stroke	602 (2.8)	29 (3.4)	.52	30 (3.6)	29 (3.4)	.89
Hypertension	9332 (43.6)	354 (41.6)	.05	348 (41.2)	351 (41.5)	.88
Diabetes	5473 (25.5)	164 (19.3)	<.001	171 (20.2)	162 (19.2)	.58
Chronic obstructive pulmonary disease	2278 (10.6)	122 (14.4)	<.001	113 (13.4)	120 (14.2)	.62
Detention	2499 (11.7)	314 (36.9)	<.001	307 (36.3)	311 (36.8)	.84
Behavioral health acuity						
None	17 778 (83.0)	613 (72.1)	<.001	631 (74.7)	609 (72.1)	.48
Mild to moderate	2303 (10.7)	104 (12.2)		93 (11.0)	104 (12.3)	
Moderate to severe	1347 (6.3)	133 (15.6)		121 (14.3)	132 (15.6)	
Psychosis disorder	2128 (9.9)	202 (23.8)	<.001	197 (23.3)	200 (23.7)	.86
Depression	8071 (37.7)	459 (54.0)	<.001	455 (53.8)	456 (54.0)	.96
Chronic pain	8703 (40.6)	388 (45.6)	.003	380 (45.0)	387 (45.8)	.73
Drug or alcohol dependence	5261 (24.6)	495 (58.2)	<.001	476 (56.3)	491 (58.1)	.46
Documented homelessness	1482 (6.9)	246 (28.9)	<.001	232 (27.5)	242 (28.6)	.59
Housing screening questions, self-reported						
Social needs based on living situation	1890 (8.8)	405 (47.6)	<.001	389 (46.0)	400 (47.3)	.59
Believe at risk of losing housing within 6 mo	2794 (13.0)	396 (46.6)	<.001	379 (44.9)	392 (46.4)	.53
Would like information about rental assistance resources	2829 (13.2)	321 (37.8)	<.001	325 (38.5)	319 (37.8%)	.76
Would like information about shelters in the area	608 (2.8)	78 (9.2)	<.001	68 (8.0)	78 (9.2%)	.39
Enrollment						
Manual enrollment reason (not automatic)	5661 (26.4)	355 (41.8)	<.001	338 (40.0)	351 (41.5)	.52
Enrollment year						
2017	8194 (38.2)	275 (32.4)	<.001	274 (32.4)	274 (32.4)	>.99
2018	5443 (25.4)	208 (24.5)		207 (24.5)	207 (24.5)	
2019	3559 (16.6)	251 (29.5)		248 (29.3)	248 (29.3)	
2020-2021	3516 (16.4)	95 (11.2)		116 (13.7)	116 (13.7)	
Preintervention health care use, mean (SD)						
Inpatient admissions	0.10 (0.49)	0.13 (0.51)	.18	0.15 (0.66)	0.13 (0.52)	.44
Emergency department visits	0.52 (1.71)	1.11 (2.27)	<.001	1.18 (3.89)	1.11 (2.27)	.64
Primary care visits	1.80 (3.00)	2.88 (5.39)	<.001	2.91 (5.36)	2.73 (4.55)	.45
Specialty care visits	1.88 (7.04)	1.46 (3.79)	.08	1.55 (5.74)	1.46 (3.80)	.70
Behavioral health visits	1.31 (7.56)	3.69 (13.81)	<.001	3.40 (14.52)	3.66 (13.82)	.71
Psychiatric emergency services	0.03 (0.48)	0.10 (0.54)	<.001	0.11 (0.88)	0.10 (0.54)	.79
Detention intakes	0.03 (0.30)	0.11 (0.47)	<.001	0.14 (0.51)	0.11 (0.48)	.35

Abbreviation: NA, not applicable.

^a^ The other or unknown category includes American Indian or Alaska Native, other low-reported categories, and missing data. This category was grouped together owing to small sample sizes.

Those who received deposits had a median (IQR) of 5 (2-11) telephonic visits and 2 (0-6) in-person visits during the year after enrollment, while the comparison group had a median (IQR) of 3 (1-6) telephonic visits and 0 (0-3) in-person visits. Participants were not matched on visits since a deposit would likely catalyze case manager interaction even before distributed, and variables affected by the treatment of interest should be avoided in the matching process.^23^

### Preintervention Health Care Use

Mean (SD) health services use among deposit recipients vs the comparison group in the 6-month preperiod was 0.13 (0.52) vs 0.15 (0.66) inpatient admissions (*P* = .44), 1.11 (2.27) vs 1.18 (3.89) emergency department visits (*P* = .63), 2.73 (4.55) vs 2.91 (5.36) primary care visits (*P* = .45), 1.46 (3.80) vs 1.55 (5.74) specialty care visits (*P* = .70), 3.67 (13.82) vs 3.40 (14.52) behavioral health visits (*P* = .71), 0.10 (0.54) vs 0.11 (0.88) psychiatric emergency services (*P* = .79), and 0.11 (0.48) vs 0.14 (0.51) detention intakes (*P* = .35) (Table 1). All services demonstrated preintervention parallel trends.

### Health Care Use Trends

**Figure 2.**
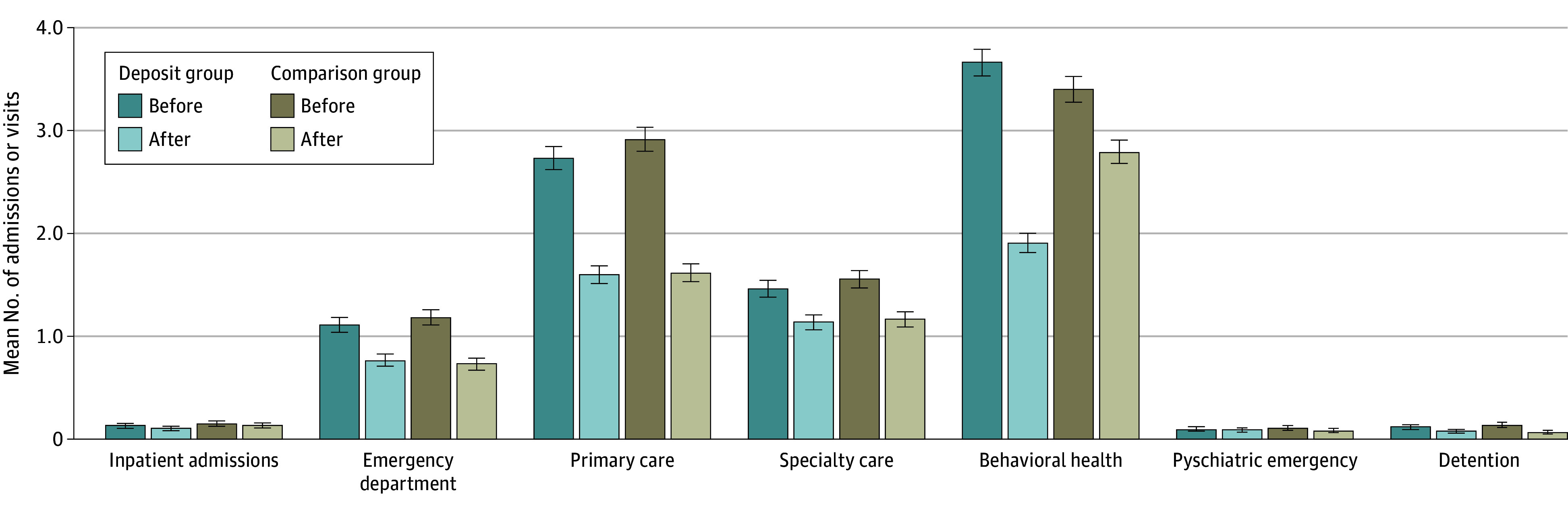
Health Care Use per Participant During the 6 Months Before and After Rental Housing Deposit Funding, Unadjusted Error bars represent 95% CIs for each mean, calculated based on Poisson distributions.

Health care use declined for all services in both the deposit group and the comparison group from 6 months preintervention to 6 months postintervention (Figure 2). Decreases in health care use were statistically significant in both groups for emergency department and primary care visits based on unadjusted linear estimates. Preintervention and postintervention decreases were also statistically significant for behavioral health visits in the deposit group and detention intakes in the comparison group. In 12-month sensitivity analyses, preintervention and postintervention decreases in health care use were statistically significant for all outcomes except psychiatric emergencies in the deposit group and for all outcomes except behavioral health visits and psychiatric emergencies in the comparison group (Table 2).

**Table 2.** Difference-in-Differences Across Health Care Use Outcomes 6 Months Before and After Intervention

Outcome	No. of visits						Marginal effects estimate (95% CI)^b^
	Deposit group			Comparison group			
	Before	After	Difference (95% CI)^a^	Before	After	Difference (95% CI)^a^	
Inpatient admissions	0.127	0.102	−0.025 (−0.071 to 0.021)	0.149	0.131	−0.018 (−0.076 to 0.040)	−0.018 (−0.088 to 0.052)
Emergency department visits	1.109	0.767	−0.342 (−0.548 to −0.136)	1.182	0.727	−0.455 (−0.770 to −0.142)	0.079 (−0.197 to 0.353)
Primary care visits	2.730	1.596	−1.134 (−1.494 to −0.774)	2.914	1.615	−1.299 (−1.749 to −0.847)	0.160 (−0.313 to 0.633)
Specialty care visits	1.460	1.134	−0.326 (−0.669 to 0.016)	1.553	1.162	−0.391 (−0.915 to 0.134)	0.200 (−0.280 to 0.680)
Behavioral health visits	3.659	1.905	−1.754 (−2.801 to −0.706)	3.399	2.792	−0.607 (−1.930 to 0.715)	−0.199 (−1.741 to 1.342)
Psychiatric emergency services	0.097	0.086	−0.011 (−0.082 to 0.061)	0.107	0.082	−0.025 (−0.099 to 0.049)	0.005 (−0.080 to 0.090)
Detention intakes	0.114	0.075	−0.039 (−0.080 to 0.002)	0.136	0.065	−0.071 (−0.115 to −0.027)	0.038 (−0.015 to 0.092)

^a^ Estimate and 95% CI based on unadjusted linear regression.

^b^ Based on negative binomial model, controlling for care manager type, age category, sex, race and ethnicity, behavioral health acuity, enrollment reason, diabetes diagnosis, hypertension diagnosis, chronic obstructive pulmonary disease diagnosis, depression diagnosis, psychosis diagnosis, alcohol and other drug dependence, detention history, homeless status from medical record documentation, and responses to housing security screening questions.

### Difference-in-Differences Outcomes

In models fully adjusted for demographics, health history, and other covariates, including baseline health care use, there was no differential change in health care use across all outcomes, including inpatient admissions, emergency department visits, primary care visits, specialty care visits, behavioral health visits, psychiatric emergency services, and detention intakes (Table 2).

### Sensitivity Analyses

Like the 6-month results, analysis of 12-month outcomes were not associated with differential use for any outcome (eTable 1 in Supplement 1). Results were also consistent in analyses with 5 matches per deposit recipient (eTable 2 in Supplement 1).

## Discussion

Several states are making large investments in Medicaid-based housing interventions, including Arkansas ($100 million), Arizona ($550 million), Oregon ($1 billion), and California ($12 billion).^24^ Amid substantial, increasing interest in new Medicaid benefits such as housing navigation, tenancy support, and housing deposits, this analysis of a Medicaid case management program for health and social needs did not find statistically significant differential reductions in health services use among housing deposit recipients relative to a matched comparison group that received case management only.

This study provides, to our knowledge, the first early evidence on housing deposits, navigation, and health care use. Thus far, the most analogous work includes studies of permanent supportive housing,^2,9,25,26,27^ where statistically significant fewer emergency department visits and primary care visits have been observed among intervention groups. Specifically, participants who moved into permanent supportive housing averaged 1.6 fewer emergency department visits and 4 fewer outpatient visits in the year after their move^25^; however, that analysis did not include a comparison group. In the more robust evaluation of New York’s Medicaid Redesign, the difference-in-differences results among permanent supportive housing participants in the year after housing were less than 1 emergency department visit and less than 1 primary care visit.^27^ Meanwhile, housing vouchers, which like deposits do not entail specific housing placements, have been associated with reduced personal health care spending^13^ and reduced stress,^11,28^ yet utilization outcomes associated with vouchers have, to our knowledge, not been studied.

One explanation for similarities in health care use across both groups is that both groups received case management, minimizing the observed treatment effect for housing deposits. Recent evidence from primary care–based housing navigation similar to the case management services in the present study found that navigation yielded 2.5 fewer primary care visits in the following year relative to a matched comparison group.^29^ It is also possible that the 6-month time frame was too short. However, results were consistent in 12-month analyses, a time frame also used in other studies.^25,27^ Future evaluations should also consider multiyear time frames given the population complexity. Larger sample sizes may also be needed to discern changes in less common outcomes like inpatient admissions, psychiatric emergency services, and detention intakes. Nevertheless, this study’s sample size was on par with other studies^25,29^ and, based on confidence interval magnitudes, was well powered to detect differences reported by other studies for emergency department and primary care use.

It is still possible that deposits support better health and well-being, despite no identifiable differences in health care use between the deposit and comparison groups. For example, deposit recipients who transitioned to stable housing may have gained improved rest, a cleaner environment for health maintenance, kitchen access to cook cost-effective nutritious meals, and the ability to leverage food benefits and similar supports. Prior literature aligns with these mechanisms and has found that housing stability supports better management of health conditions and more consistent receipt of social services benefits.^30,31,32,33^

Future studies should confirm that housing deposits do not detrimentally impact access or care continuity due to relocation and potential isolation.^34^ Care disruptions are especially important to mitigate given that those who move due to financial difficulty report greater likelihood of postponing needed medical care and increased emergency department use.^35^ We anticipate that case managers helped minimize disruptions since, as health system employees, they could efficiently make care connections throughout a housing transition. Medication fill data could be one way to assess continuous access to health services and ability to manage health conditions.^36^

### Limitations

This analysis has certain limitations. Data were from a single county-based health system in an area with limited affordable housing available. Results may differ in areas with greater housing availability. Second, the intervention overlapped with the COVID-19 pandemic, in which routine health care utilization broadly declined,^37^ though temporal trends should have affected both groups similarly under the difference-in-differences study design. Third, despite robust electronic health records and integrated case management documentation, certain unobservable characteristics could not be accounted for, such as contemporaneous measures of housing stock, individual willingness to relocate, and income stability. Last, though study outcomes included a broader range of health care use measures than past research,^38,39^ available data does not include whether deposit recipients successfully retained housing. Housing retention could be an important process metric to more comprehensively understand program impact.

## Conclusions

Health care systems increasingly emphasize social determinants of health, yet evidence on new, policy-driven interventions remains limited. In this cohort study, we leveraged data from a Medicaid 1115 demonstration program to identify the association between housing deposits and health care use, building on well-documented associations between housing stability and health care use. This analysis did not find differential changes in health care use among participants who received housing deposit funding compared to a matched group receiving the same case management services without deposit funding. Nevertheless, deposit funding may have influenced case manager effectiveness and participant well-being in ways not captured by study outcomes. As Medicaid programs across the country make substantial investments in housing and health interventions, future evaluations could benefit from longer time horizons and integration with patient-centered or process metrics. Amid rapid policy changes, this work can help ascertain how to better support people experiencing long-term homelessness or housing instability, thereby improving population health.

## Notes

Supplement 1. **eFigure 1.** Average deposit lag times by enrollment start date quarter **eFigure 2.** Trend graphs for outcomes before and after housing deposit intervention **eTable 1.** Results for 12 months pre-post rental housing deposit intervention difference-in-differences **eTable 2.** Results using 5 matches per deposit recipient, with replacement

Supplement 2. Data Sharing Statement
