# Supplementary material for: Rental Housing Deposits and Health Care Use
Source: JAMA Health Forum. 2024 Sep 6;5(9):e242802. doi: 10.1001/jamahealthforum.2024.2802 (PMC11380099; doi:10.1001/jamahealthforum.2024.2802)
Supplement: Supplement 1. — eFigure 1. Average deposit lag times by enrollment start date quarter eFigure 2. Trend graphs for outcomes before and after housing deposit intervention eTable 1. Results for 12 months pre-post rental housing deposit intervention difference-in-differences eTable 2. Results using 5 matches per deposit recipient, with replacement [file jamahealthforum-e242802-s001.pdf]

Supplemental Online Content

Knox MJ, Hernandez EA, Ahern J, et al. Rental housing deposits and health care use. *JAMA Health Forum*. 2024;5(9):e242802. doi:10.1001/jamahealthforum.2024.2802

eFigure 1. Average deposit lag times by enrollment start date quarter
eFigure 2. Trend graphs for outcomes before and after housing deposit intervention
eTable 1. Results for 12 months pre-post rental housing deposit intervention difference-in-differences
eTable 2. Results using 5 matches per deposit recipient, with replacement

This supplemental material has been provided by the authors to give readers additional information about their work.

© 2024 Knox MJ et al. *JAMA Health Forum.*

eFigure 1. Average deposit lag times by enrollment start date quarter

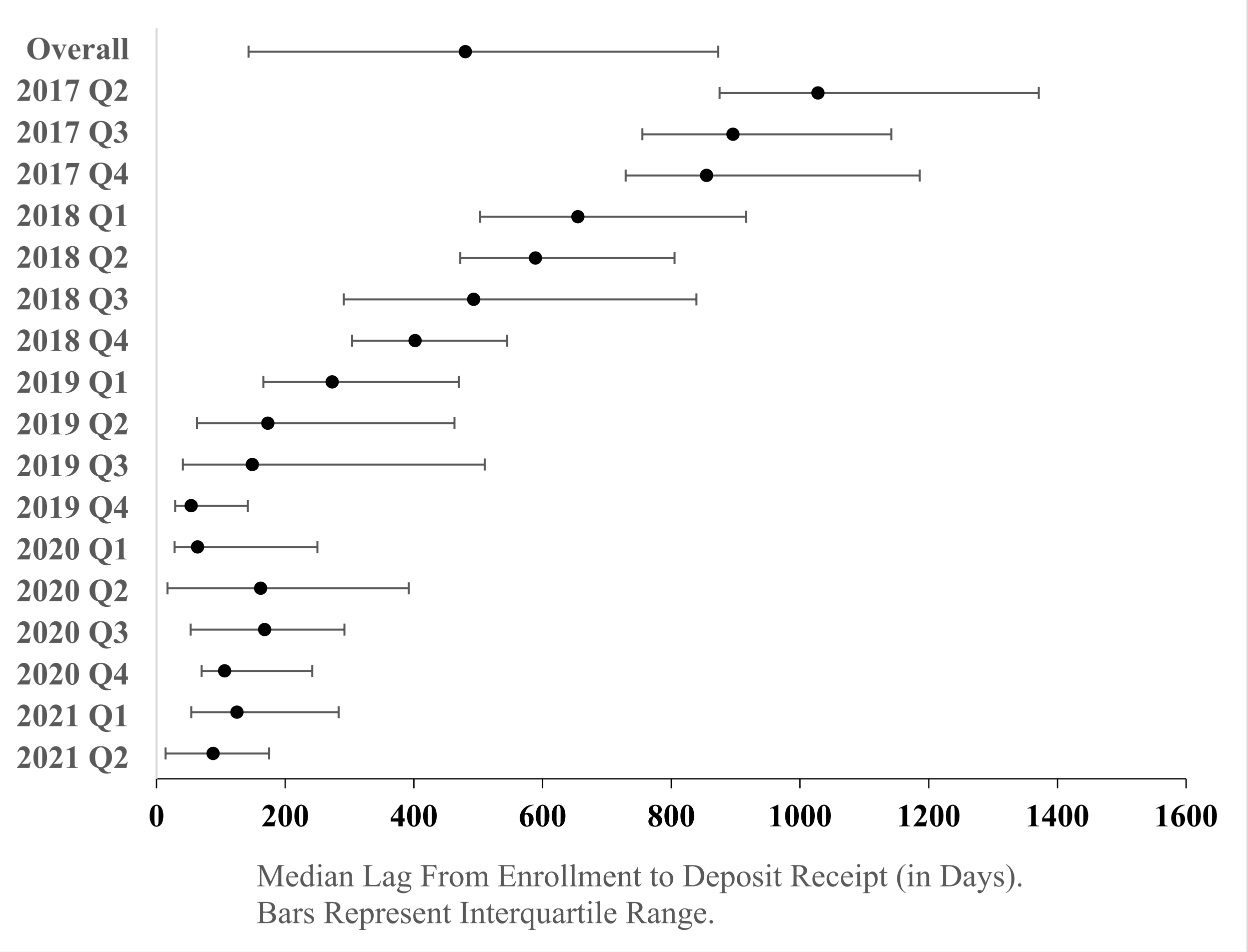

© 2024 Knox MJ et al. *JAMA Health Forum.*

eFigure 2. Trend graphs for outcomes before and after housing deposit intervention

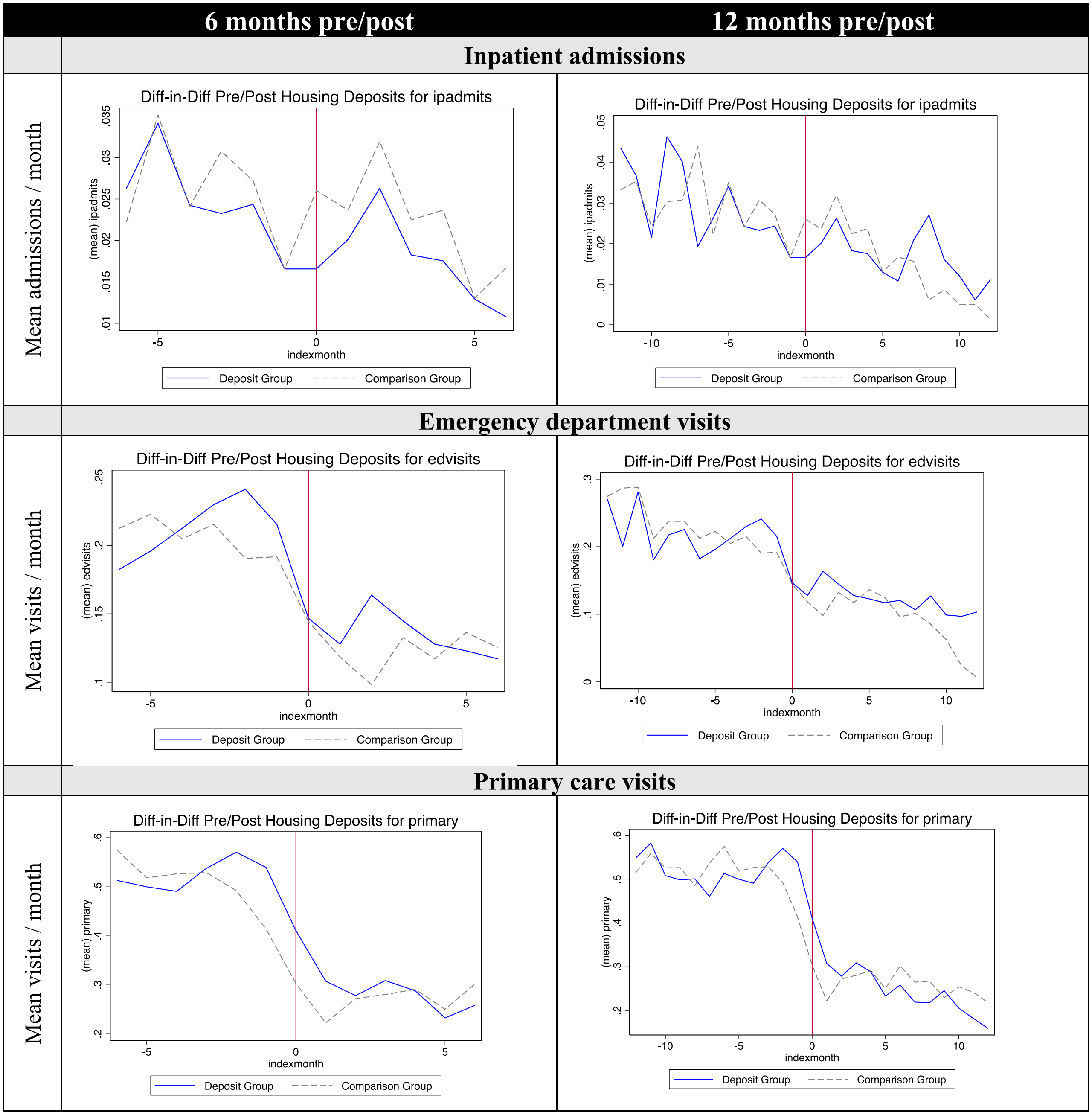

© 2024 Knox MJ et al. *JAMA Health Forum.*

Specialty care visits

Mean visits / month

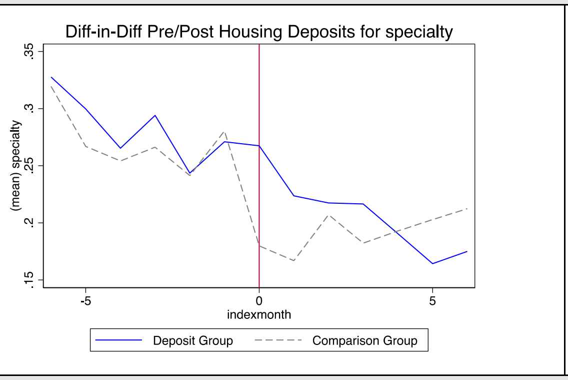

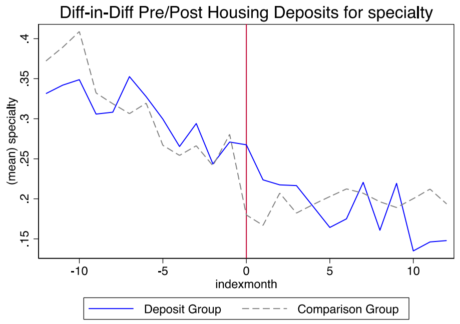

Behavioral health visits

Mean visits / month

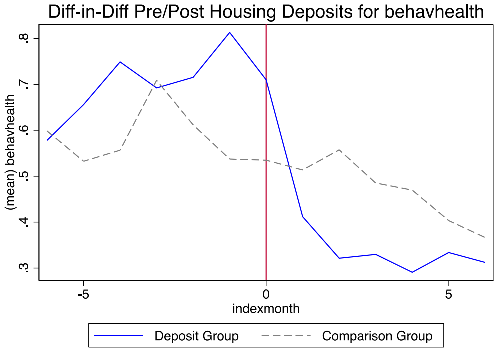

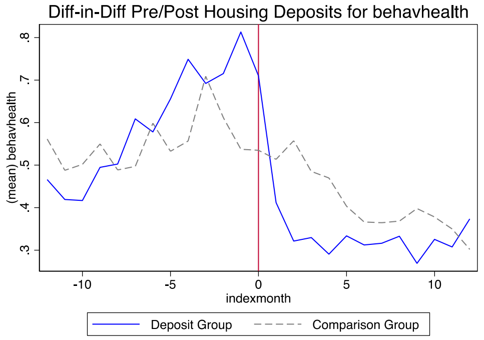

Detention intakes

Mean intakes / month

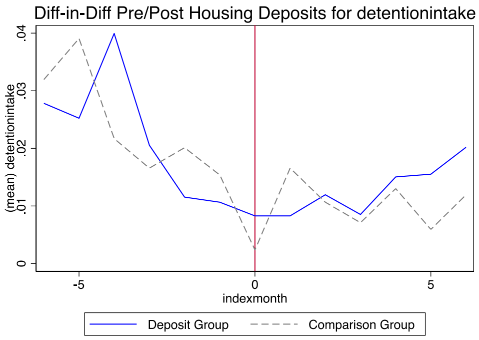

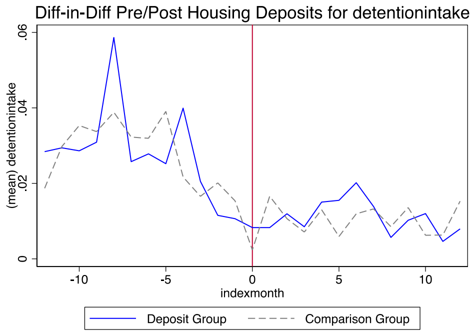

Psychiatric emergency services visits

Mean visits / month

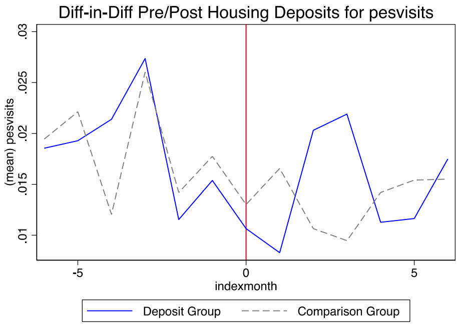

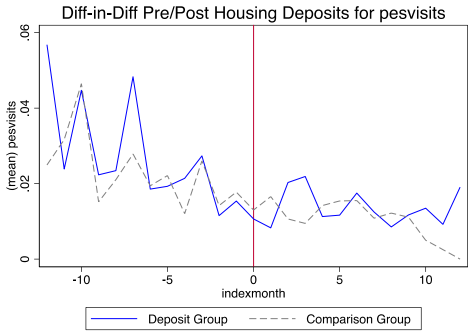

© 2024 Knox MJ et al. *JAMA Health Forum.*

eTable 1. Results for 12 months pre-post rental housing deposit intervention difference-in-differences

Mean Healthcare Use (12-months)							
	Deposit Group (# visits)			Comparison Group (# visits)			Marginal Effect**
Outcome	Before	After	Difference [95% CI]*	Before	After	Difference [95% CI]*	Estimate [95% CI]
Inpatient admits	0.266	0.178	-0.088 [-0.164, -0.014]	0.285	0.172	-0.113 [-0.197, -0.030]	0.026 [-0.074, 0.126]
ED visits	2.037	1.291	-0.746 [-1.120, -0.371]	2.219	1.089	-1.130 [-1.695, -0.565]	0.224 [-0.247, 0.693]
Primary Care	4.820	2.583	-2.237 [-2.790, -1.688]	5.054	3.024	-2.030 [-2.833, -1.229]	-0.106 [-0.889, 0.676]
Specialty Care	2.806	1.962	-0.844 [-1.430, -0.258]	2.986	2.305	-0.681 [-1.430, -0.258]	0.127 [-0.757, 1.011]
Behavioral Health	5.639	3.441	-2.198 [-3.946, -0.450]	5.492	4.854	-0.638 [-2.675, 1.400]	-0.572 [-3.056, 1.912]
Psychiatric Emergency	0.245	0.146	-0.099 [-0.260, 0.061]	0.219	0.122	-0.097 [-0.220, 0.025]	-0.039 [-0.179, 0.101]
Detention	0.251	0.118	-0.133 [-0.214, -0.051]	0.266	0.125	-0.141 [-0.214, -0.051]	0.010 [-0.098, 0.119]

IP = inpatient; ED = emergency department; CI = confidence interval

* = Based on unadjusted linear regression
** = Based on negative binomial model, controlling for care manager type, age category, sex, race, behavioral health acuity, enrollment reason, diabetes dx, hypertension dx, copd dx, depression dx, psychosis dx, alcohol and other drug dependence, detention history, homeless status from medical record documentation, and responses to housing security screening questions

© 2024 Knox MJ et al. *JAMA Health Forum.*

eTable 2. Results using 5 matches per deposit recipient, with replacement

Mean Healthcare Use (6-months)							
	Deposit Group (# visits)			Comparison Group (# visits)			Marginal Effect**
Outcome	Before	After	Difference [95% CI]*	Before	After	Difference [95% CI]*	Estimate [95% CI]
Inpatient admits	0.126	0.102	-0.024 [-0.070, 0.022]	0.133	0.122	-0.011 [-0.047, 0.025]	-0.018 [-0.074, 0.039]
ED visits	1.101	0.767	-0.334 [-0.540, -0.129]	1.070	0.738	-0.332 [-0.047, 0.025]	-0.002 [-0.231, 0.227]
Primary Care	2.712	1.596	-1.116 [-1.474, -0.757]	2.742	1.528	-1.214 [-1.516, -0.912]	0.079 [-0.318, 0.476]
Specialty Care	1.456	1.134	-0.322 [-0.664, 0.019]	1.466	1.382	-0.084 [-0.398, 0.229]	-0.217 [-0.660, 0.226]
Behavioral Health	3.633	1.905	-1.728 [-2.770, -0.686]	3.244	2.410	-0.834 [-1.803 ,0.134]	0.281 [-0.904, 1.465]
Psychiatric Emergency	0.096	0.086	-0.010 [-0.081, 0.061]	0.101	0.108	0.007 [-0.055, 0.070]	-0.012 [-0.088, 0.064]
Detention	0.113	0.075	-0.038 [-0.079, 0.003]	0.129	0.088	-0.041 [-0.092, 0.009]	0.019 [-0.035, 0.072]

Mean Healthcare Use (12-months)							
	Deposit Group (# visits)			Comparison Group (# visits)			Marginal Effect**
Outcome	Before	After	Difference [95% CI]*	Before	After	Difference [95% CI]*	Estimate [95% CI]
Inpatient admits	0.264	0.178	-0.086 [-0.161, -0.012]	0.273	0.164	-0.109 [-0.162, -0.056]	0.031 [-0.049, 0.111]
ED visits	2.024	1.291	-0.733 [-1.105, -0.360]	2.102	1.103	-0.999 [-1.437, -0.560]	0.080 [-.319, 0.478]
Primary Care	4.818	2.583	-2.235 [-2.781, -1.688]	4.782	2.883	-1.899 [-2.381, -1.416]	-0.274 [-0.918, 0.371]
Specialty Care	2.805	1.962	-0.843 [-1.426, -0.259]	2.783	2.677	-0.106 [-1.426, -0.259]	-0.409 [-1.233, 0.415]
Behavioral Health	5.599	3.441	-2.158 [-3.898, -0.418]	5.555	4.249	-1.306 [-2.862, 0.251]	-0.017 [-1.962, 1.928]
Psychiatric Emergency	0.243	0.146	-0.097 [-0.257, 0.062]	0.226	0.145	-0.081 [-0.190, 0.029]	-0.052 [-0.179, 0.075]
Detention	0.249	0.118	-0.131 [-0.212, -0.050]	0.253	0.141	-0.112 [-0.185, -0.038]	-0.028 [-0.128, 0.071]

* = Based on unadjusted linear regression ** = Based on negative binomial model, controlling for care manager type, age category, sex, race, behavioral health acuity, enrollment reason, diabetes dx, hypertension dx, copd dx, depression dx, psychosis dx, alcohol and other drug dependence, detention history, homeless status from medical record documentation, and responses to housing security screening questions

© 2024 Knox MJ et al. *JAMA Health Forum.*
