# Supplementary material for: Rental Housing Deposits and Health Care Use
Source: JAMA Health Forum. 2024 Sep 6;5(9):e242802. doi: 10.1001/jamahealthforum.2024.2802 (PMC11380099; doi:10.1001/jamahealthforum.2024.2802)
Supplement: Supplement 2. — Data Sharing Statement [file jamahealthforum-e242802-s002.pdf]

Data Sharing Statement

Knox. Rental Housing Deposits and Health Care Use. *JAMA Health Forum*. Published September 06, 2024. doi:10.1001/jamahealthforum.2024.2802

Data
Data available: No

Additional Information
Explanation for why data not available: Data are not publicly available. Please contact the corresponding author for further information.
